# Enhanced neuroprotective efficacy of bone marrow mesenchymal stem cells co-overexpressing BDNF and VEGF in a rat model of cardiac arrest-induced global cerebral ischemia

**DOI:** 10.1038/cddis.2017.184

**Published:** 2017-05-11

**Authors:** Lili Zhou, Qingming Lin, Peng Wang, Lan Yao, Kahong Leong, Zhiqun Tan, Zitong Huang

**Affiliations:** 1 Department of Emergency Medicine, Sun Yat-sen Memorial Hospital, Sun Yat-sen University, Guangzhou 510120, China; 2 Department of Emergency Medicine, Institute of Cardiopulmonary Cerebral Resuscitation, Sun Yat-sen University, Guangzhou 510120, China; 3 Department of Emergency Medicine, Fujian Provincial Hospital, Fujian Medical University, Fuzhou 350001, China; 4 Department of Emergency Medicine, The Fifth Affiliated Hospital, Sun Yat-sen University, Zhuhai 519000, China; 5 Department of Emergency Medicine, Hospital Conde S. Januario, Macau, China; 6 Institute for Memory Impairments and Neurological Disorders, University of California Irvine, Irvine, CA 92697, USA; 7 Institute of Precision Medicine, Jining Medical University, Jining 272067, China

## Abstract

Cardiac arrest-induced global cerebral ischemia injury (CA-GCII) usually leads to a poor neurological outcome without an effective treatment. Bone marrow-derived mesenchymal stem cells (BMMSCs) may provide a potential cell-based therapy against neurologic disorders through induction of brain-derived neurotrophic factor (BDNF) and vascular endothelial growth factor (VEGF). To optimize the neuroprotective efficacy of BMMSCs further, in this study we have derived BMMSCs, which co-overexpress both BDNF and VEGF, and tested them for the treatment of CA-GCII in a rat model. Lentiviruses that express rat BDNF exon IV or VEGF-A were created using the bicistronic shuttle vectors of pLVX-IRES-ZsGreen1 and pLVX-IRES-tdTomato, respectively. BMMSCs that were co-transduced with the engineered lentiviruses with co-overexpression of both BDNF and VEGF along with corresponding fluorescent protein reporters were injected via jugular vein of rats that just recovered from a cardiac arrest. Animals were then scored for neurofunctional deficits and examined for brain pathology and gene expression relevant to the engraftment seven days after the treatments. We demonstrate that anchorage of lentiviral vector-transduced BMMSCs, which co-overexpressed both BDNF and VEGF in the hippocampus and temporal cortex along with significantly ameliorated brain pathology and improved neurofunctional performance in CA-GCII rats after transplantation. These findings provide a proof of concept for the further validation of engineered BMMSCs for the treatment of CA-GCII patients in clinical practice in the future.

Cardiac arrest (CA) is associated with both high morbidity and mortality rates and poses the most serious threat to human life.^1^ In recent years, with the continuous improvement in the emergency network and broad application of cardiopulmonary resuscitation and defibrillation technology, there is an increase in the success rate of resuscitation of out-of-hospital CA patients with restoration of spontaneous circulation (ROSC).^2^ However, many of these patients usually suffer from complicated neural dysfunction or even death as a sequel to brain damage due to global cerebral ischemia following CA.^3^ Despite the use of neuroprotective medicines and hypothermic treatments, which to some extent may ameliorate brain injury in clinic,^4^ there is still an urgent demand for new treatments to improve the prognosis of CA-induced brain injury. In this regard, stem cells offer exciting promise for rebuilding the nervous system to treat devastating brain disorders including global cerebral ischemia resulting from CA. Because of the multipotency to divide and to differentiate into functional neural cells,^5^ bone marrow-derived mesenchymal stem cells (BMMSCs) have been widely tested for the treatment of a broad spectrum of degenerative disorders, such as cardiovascular diseases and neurologic complications.^6, 7, 8, 9^ We and others previously showed the therapeutic benefit of BMMSCs in CA-induced global cerebral ischemic injury (CA-GCII).^10, 11^ Transplantation of BMMSCs significantly improved neural functional recovery of rats from CA-GCII. Mechanistic analysis suggests that the therapeutic efficacy of stem cells transplanted might be mediated by secreted vascular endothelial growth factor (VEGF) and brain-derived neurotrophic factor (BDNF).^12, 13^ Therefore, to optimize the efficacy further, in this study we employed lentiviral vectors to induce overexpression of both BDNF and VEGF in BMMSCs, thereafter examined the neuroprotective efficacy of the virus-transduced BMMSCs for treatments of brain lesions and neurofunctional deficits after CA-GCII in rats. The results indicate markedly enhanced neuroprotective potency of BMMSCs for CA-induced global cerebral ischemia.

## Results

### Lentivirus-mediated overexpression of BDNF and VEGF in rat BMMSCs

**Figure 1:**
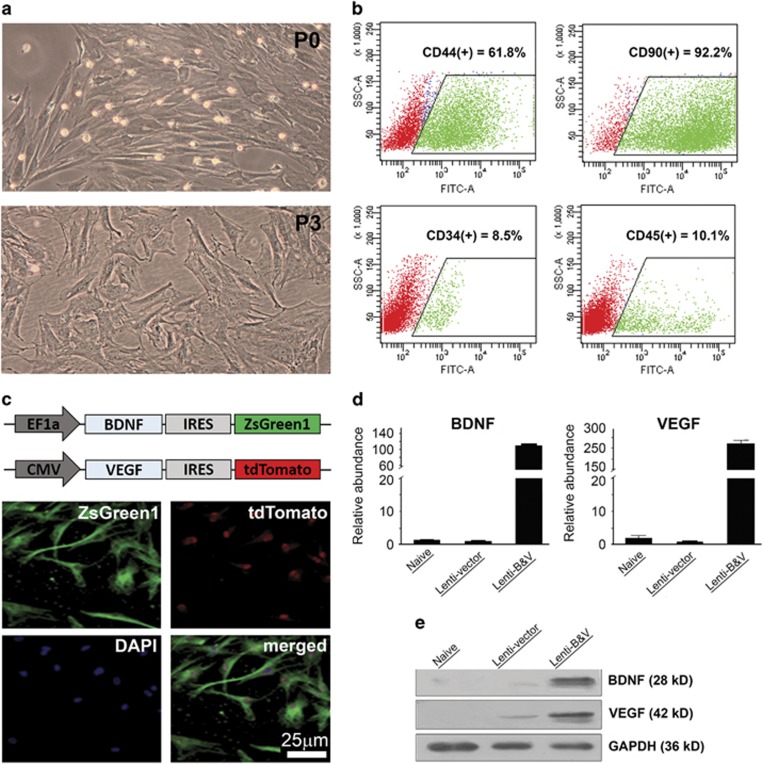
Characterization of cultured rat BMMSCs and detection of lentivirus-mediated overexpression of BDNF and VEGF. (**a**) Representative phase-contrast micrographs show the morphology of rat BMMSC cultures at passage 0 (P0) and passage 3 (P3); (**b**) Flow cytometry analysis shows the majority of BMMSCs at P3-expressing stem cells markers CD44 and CD90, and a small portion of cells expressing CD34 and CD44; (**c**) Schematics (on the top) show the expression cassette with either rat brain BDNF exon IV or VEGF-A open reading frame coding sequence followed by an IRES-directed ZsGreen1 and tdTomato fluorescent protein coding sequences in the lentivirus constructs, respectively; fluorescence micrographs (four panels in the bottom) show that transduced BMMSCs co-express both ZsGreen1 (green) and tdTomato (red) fluorescent proteins; (**d**) RT-qPCR results show the relative abundance of BDNF and VEGF mRNAs in un-transduced (naive) BMMSCs as well as 48 h after transduction with an empty lentiviral vector (Lent-vector) or BDNF-&VEGF lentivirus-co-transduced (Lenti-B&V). Data are depicted as mean±S.D.; (**e**) Western blots of whole-cell lysates detect markedly increased levels of BDNF and VEGF in cells co-transduced with both BDNF- and VEGF-lentiviruses

Once MSCs were prepared from rat bone marrow and grown in flasks, the identity of these cells was identified by both morphology and specific molecular markers. Phase-contrast microscopy demonstrated their adherence onto the surface of plastic substrates with a fibroblast-like appearance (Figure 1a), whereas flow cytometry analysis revealed that both CD44 and CD90, two specific surface markers for MSC, were positive in the majority (61.8 and 91.2%, respectively) of cells after three passages, and only a small portion of cells showed expression of blood cell-related markers, CD34 and C45 (Figure 1b). Cells were co-transduced with the lentivirus constructs carrying an expression cassette for rat BDNF or VEGF along with ZsGreen1 or tdTomato fluorescent protein, respectively. Fluorescence microscopy detected expression of both fluorescent proteins in most cells, indicating the success of transduction at a high efficiency (Figure 1c), as ZsGreen1 and tdTomato open reading frames were directed by an internal ribosome entry site (IRES) within either BDNF or VEGF cassette. It is notable that both ZsGreen1 and tdTomato proteins exhibit stronger fluorescence in the nucleus or perinuclear area than the cytoplasm as reported.^14, 15^ RT-qPCR using specific primers for BDNF or VEGF confirmed a dramatic increase in expression of both BDNF and VEGF transcripts in the cells co-transduced by the lentiviruses carrying both genes compared with cells either un-transduced or transduced with the viruses without these two genes (Figure 1d). These observations were consistent with the results from Western blots of cell lysates using a specific BDNF or VEGF antibody, in which glyceraldehyde 3-phosphate dehydrogenase (GAPDH) immunoreactivity was used as a loading control (Figure 1e).

### BDNF and VEGF expression in the brain of CA-GCII rats after transplantation of BMMSCs

**Figure 2:**
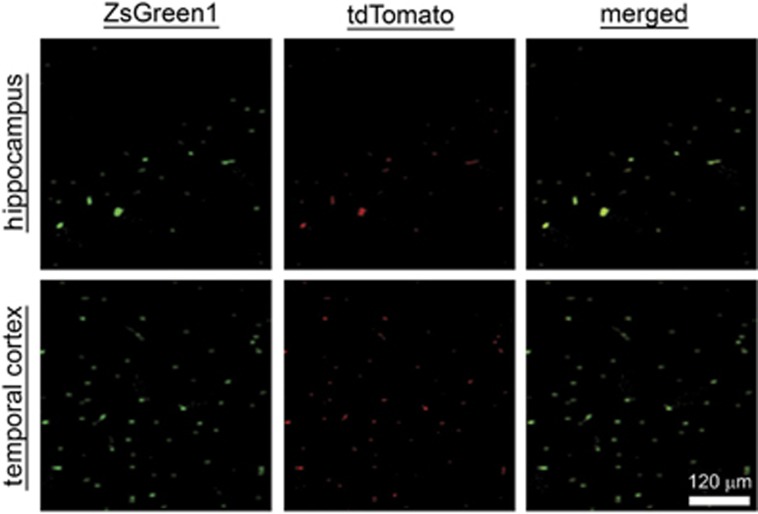
Distribution of lentivirus-transduced BMMSCs in hippocampus and temporal cortex areas of rat brain after transplantation. Representative fluorescent micrographs show the co-expression of both ZsGreen1 (green) and tdTomato (red) fluorescent proteins in the hippocampal CA1 (top row) and temporal cortex areas (bottom row) of a coronal section from the rat on day 7 after CA-GCII with BM-B&V transplantation

**Figure 3:**
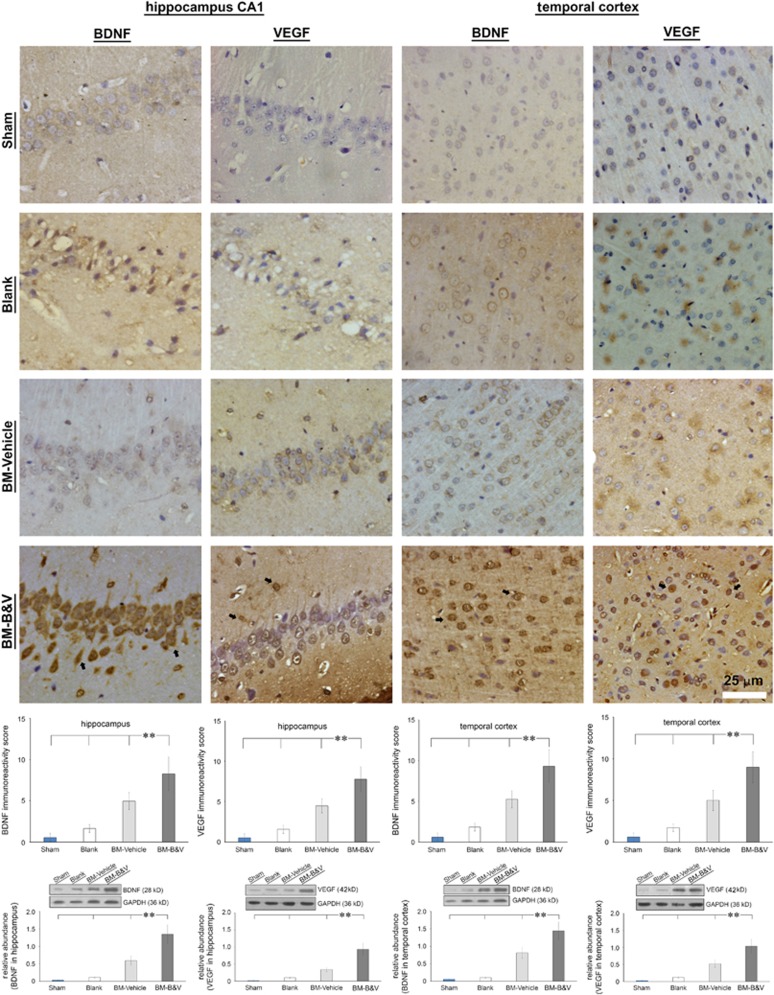
Transplantation of lentivirus-transduced BMMSCs boosted levels of BDNF and VEGF proteins in the rat brain. Immunohistochemistry using a specific antibody against BDNF or VEGF was performed on coronal sections from each animal in the respective groups. Representative micrographs demonstrate differential immunoreactivity of both BDNF and VEGF in both hippocampal CA1 and temporal cortex areas of rat brains from respective groups. Arrows indicate typical immunoreactive cells. Quantified BDNF- or VEGF-immunoreactive cells in the hippocampus or temporal cortex are depicted as immunoreactivity scores (the last second row). Western blots of tissue lysates from hippocampus or temporal cortex and corresponding densitometric quantification relative to the GAPDH loading control confirm immunohistochemistry results (bottom row). Data are depicted as mean±S.D. ** indicates *P*-value <0.001. Sham, normal control; Blank, CA-GCII with PBS; BM-Vehicle, CA-GCII with naïve BMMSCs; BM-B&V, CA-GCII with BMMSCs co-transduced with BDNF- and VEGF-lentiviruses

Normally the abundance of both BDNF and VEGF is relatively low in the rat brain, increased expression of these two factors was found in the hippocampus and temporal cortex after ROSC.^16, 17^ Growing studies demonstrate protective efficacy of both BDNF and VEFG for a variety of ischemic damage in the brain.^18, 19^ Considering the advantage of neural stem cells for the treatment of brain damage,^20^ we previously showed therapeutic benefits of transplantation of naive BMMSCs for suppressing CA-GCII in rats.^11, 21, 22^ The observations from us as well as other groups suggest that increased expression of both BDNF and VEGF by the transplanted BMMSCs, at least in part, contribute to their neuroprotective activities.^11, 23^ Furthermore, in our studies we found that, although direct lateral ventricular administration of BMMSCs provided highest efficiency to deliver the cells into the injured brain and to demonstrate the benefits,^11^ the operational procedures were invasive and more complicated than the venous approach. Despite a high proportion of pulmonary retention,^24^ certain number of BMMSCs administered intravenously still successfully migrated into lesioned zones of the brain with CA-GCII, particularly in the hippocampus and cortex.^10, 21^Accordingly, here we employed BMMSCs with lentivirus-directed co-overexpression of BDNF and VEGF to optimize the therapeutic benefits by delivering the engineered cells to the rat brain via the jugular vein injection after ROSC from CA-GCII. Then we specially focused on the changes occurring in the hippocampus and temporal cortex, two areas that are the most vulnerable to the CA-GCII and enriched most engrafted BMMSCs as characterized previously.^10^ After cell transplantation, fluorescence microscopy detected expression of both ZsGreen1 and tdTomato fluorescence proteins in the hippocampus and temporal cortex (Figure 2). These observations confirmed anchorage of the transplanted cells in these areas in the brain, which was consistent with our previous findings.^10, 11^ Importantly, differential increases in the immunoreactivity of BDNF and VEGF were shown by immunohistochemistry in the same regions in the group transplanted with BDNF&VEGF-BMMSCs (i.e., BM-B&V) or naive BMMSCs (i.e., BM-Vehicle) in contrast to the group given PBS (Blank) or sham operated (Sham) (Figure 3), but the BM-B&V group demonstrated significantly more cells with higher intensity of immunostaining compared to all the other groups. Meanwhile, although the exactly same staining conditions were applied for all the brain sections, these from the BM-B&V group constantly exhibited higher background in both BDNF and VEGF staining than those from the other groups. These findings were further corroborated by immunoreactivity scores-based cell counting (Figure 3, the second-row panels to the bottom) and Western blotting analysis of brain lysates (Figure 3, the bottom row).

### BMMSC-directed overexpression of BDNF and VEGF ameliorates brain damage in CA-GCII rats

**Figure 4:**
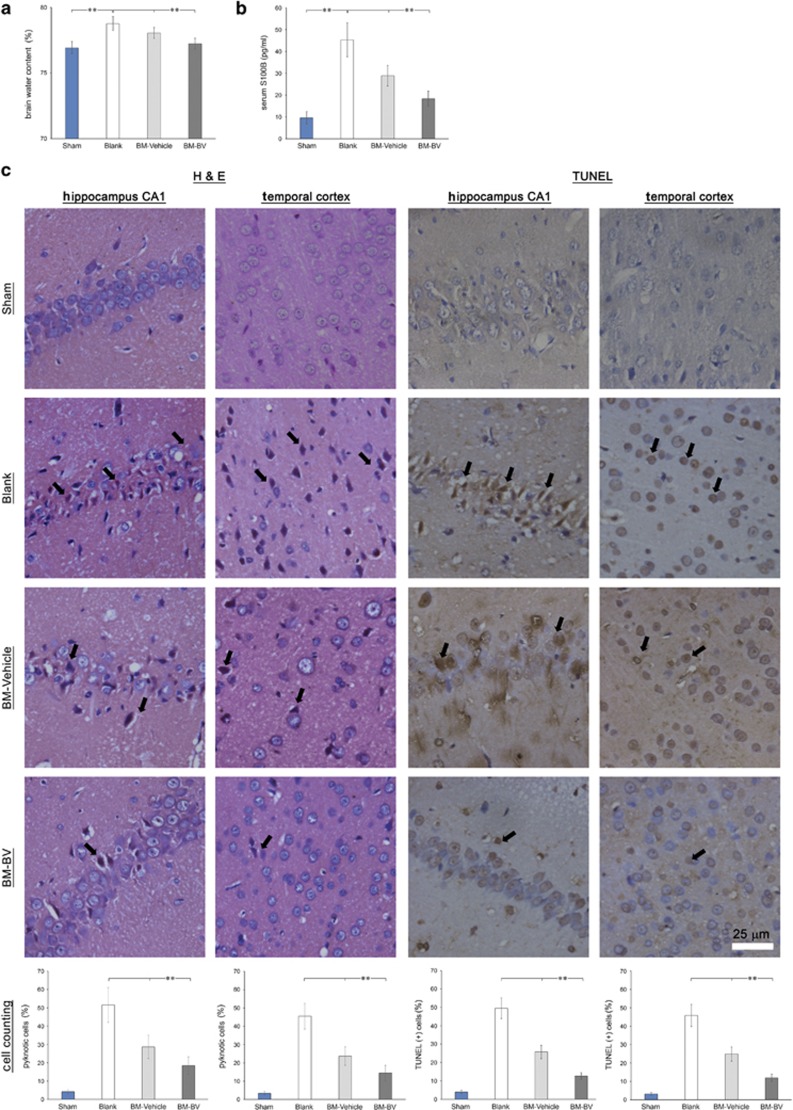
Transplantation of lentivirus-transduced BMMSCs ameliorated brain damage indicated by the reduction of brain edema, serum S100B levels, pyknotic cells in H&E staining, and TUNEL-positive cells. (**a**) Changes in brain water content were measured by weighting from five animals in each group (*N*=5); (**b**) Levels of serum S100B from each animal were determined by ELISA; (**c** and **d**) Representative micrographs from H&E stained (**c**) or TUNEL-labeled (**d**) coronal sections show the differential brain damage in both hippocampal CA1 and temporal cortex areas of rat brains from respective groups. Arrows indicate pyknotic/eosinophilic cells (**c**) and TUNEL-positive cells (**d**) in both hippocampal CA1 and temporal cortex areas in coronal sections from rats with or without CA-GCII following the different treatments. Quantifications of damaged cells are depicted as percentage *versus* total number of cells within the respective microscopic fields (mean±S.D.). ** indicates *P*-value <0.001. Sham, normal control; Blank, CA-GCII with PBS; BM-Vehicle, CA-GCII with naïve BMMSCs; BM-B&V, CA-GCII with BMMSCs co-transduced with BDNF- and VEGF-lentiviruses

The purpose of transplantation of BMMSCs was to test whether transplanted cells can protect brain cells from CA-induced ischemic damage, particularly whether the modified BMMSCs that overexpress BDNF and VEGF deliver greater potency compared to the naive cells, we therefore sought to determine changes in the severity of brain damage after CA-GCII with different engraftment. In animal models, a change in brain water content (water content/wet weight, %) has been used as a simple indicator for brain edema.^25^ Figure 4a shows the significantly higher level of water content in the CA-GCII rat brain compared to the sham control. However, transplantation of BMMSCs overexpressing both BDNF and VEGF dramatically reduced brain water content and made it close to that in the sham control. Serum S100B levels have also been reported as an informative surrogate biomarker for ischemia-related brain damage.^26, 27^ Strikingly, ELISA-measured S100B level in the sera from the blood collected on postoperative day 7 showed a significant decrease in BM-B&V group compared to both BM-Vehicle and PBS blank controls, although it was still slightly higher than the sham control (Figure 4b). Hematoxylin & eosin (H&E) staining demonstrated the normal morphology and no damaged cells in hippocampal CA1 and temporal cortex areas in the brain from the sham control, but massive damaged cells with notable neural cell loss in the corresponding areas in the brains from both PBS blank and BM-Vehicle controls after CA-GCII. As previously reported,^28^ damaged brain cells appeared shrunken with eosinophilic perikaryons, vacuolization, and nuclear pyknosis under light microscope. Despite the CA-GCII challenge, the BM-B&V group that was engrafted BMMSCs co-overexpressing BDNF and VEGF showed well-preserved morphology with very few damaged cells in the corresponding areas. Quantification of damaged cells in both CA1 and temporal cortex areas confirmed significant protection efficacy of BM-B&V engraftment (Figure 4c, left two columns). This result was further supported by the examinations from TUNEL staining that demonstrated degenerating cells in the respective brain regions (Figure 4c, right two columns).

### BMMSC transplantation stimulates angiogenesis in the brain of CA-GCII rats

**Figure 5:**
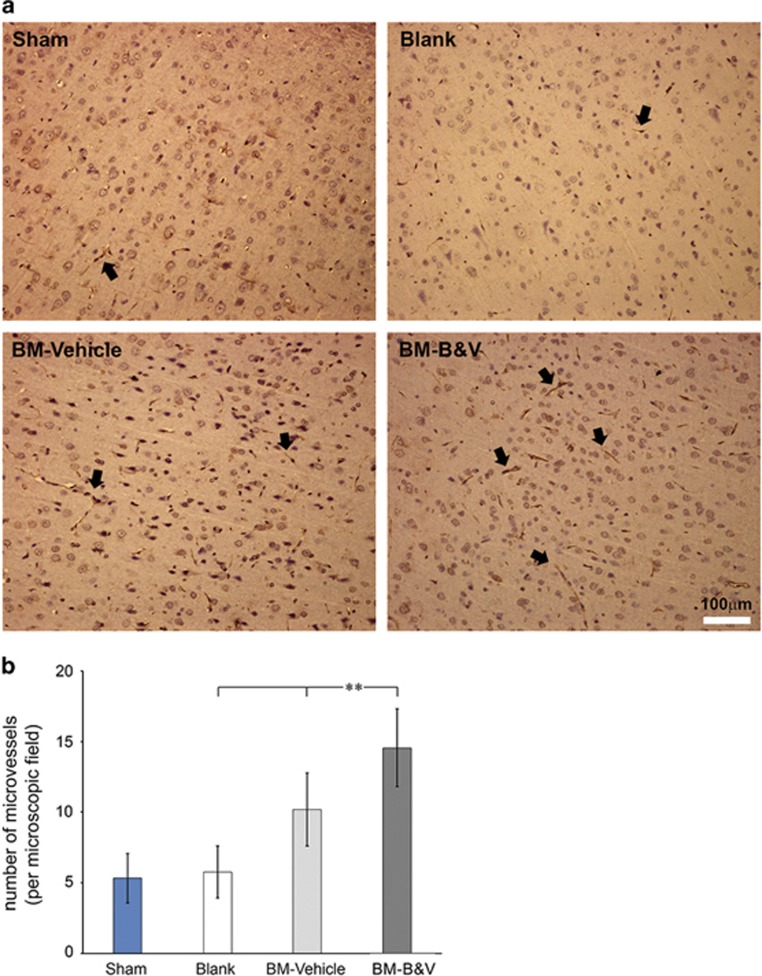
Immunohistochemistry detects increased angiogenesis in the rat brain with CA-GCII by transplanted BMMSCs. (**a**) Microscopic images from temporal cortex area in coronal sections stained with anti-RECA-1 antibody. Arrows indicate RECA-1-positive microvessels. (**b**) Quantification of microvessels is expressed as microvessel density (number of stained vessels per microscopic field). Data are depicted as mean±S.D. ** indicates *P*-value <0.001 for Student’s *t*-test. Sham, normal control; Blank, CA-GCII with PBS; BM-Vehicle, CA-GCII with naïve BMMSCs; BM-B&V, CA-GCII with BMMSCs co-transduced with BDNF- and VEGF-lentiviruses

Converging evidence suggests that both BDNF and VEGF promote angiogenesis in the brain thereby contribute to the protection of brain cells from ischemic injury.^29, 30, 31^ Therefore, we next asked whether the brain protection was associated with any increased angiogenesis in brains after BMMSC transplantation. Immunohistochemistry using a specific antibody against RECA-1, a specific cell surface marker for rat vascular endothelial cells, showed a significant increase in the density of microvessels in the temporal cortex in CA-GCII rats from the BM-B&V group in comparison with the other groups (Figure 5).

### BMMSC-mediated brain protection is associated with neurofunctional improvement

**Figure 6:**
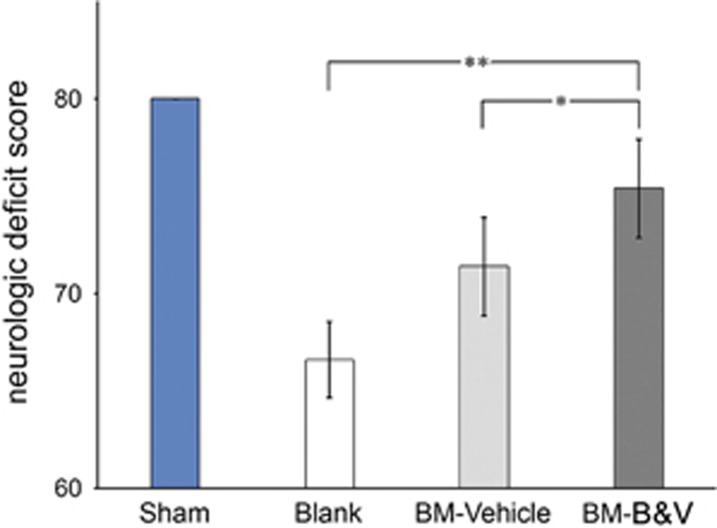
Neurofunctional assessment shows improvement of neurologic deficit score in CA-GCII rats by transplanted BMMSCs. A comprehensive NDS for all the rats was examined at day 7 after CA-GCII and cell transplantation as described in the Materials and Methods section. Data are scaled to the mean±S.D. *P <0.05 and **P <0.01 for the Wilcoxon–Mann–Whitney test. Sham, normal control; Blank, CA-GCII with PBS; BM-Vehicle, CA-GCII with naive BMMSCs; BM-B&V, CA-GCII with BMMSCs co-transduced with BDNF- and VEGF-lentiviruses

Neurological deficit is one of the major neuro-dysfunctional expectations of global cerebral ischemia. Neurological deficits of all the rats were scored using Geocadin’s method right before killing. Figure 6 depicts the NDS values of all four groups of animals. Obviously, the sham control rats exhibited the highest/full score of 80, whereas PBS-treated CA-GCII rats (Blank) showed the lowest score of 66±1.95. Although both BMMSC-transplanted groups had higher scores than the Blank, only the BM-B&V group demonstrated significant improvement in this score compared to the blank control, which is in agreement with all the histopathological findings.

## Discussion

Brain injury after transient global cerebral ischemia from CA is associated with significant morbidity and mortality, and has long-term neurologic and neurosensory sequelae in survivors. Growing studies have demonstrated promises of stem cell therapy for brain repair against brain damage resulting from various insults.^32, 33^ In addition to migrating directly into the lesion sites to differentiate into neuronal and glial cells to replace dead or damaged cells for the reconstruction of impaired neural circuits,^34, 35^ transplanted stem cells are a by-stander secreting neurotrophic factors, antioxidant molecules, and anti-inflammatory cytokines, and subsequently promote sparing of host cells and stimulate neurogenesis.^23, 36^ In this study, naive BMMSC engraftment exhibited moderate benefits for the rats after CA-GCII. Direct fluorescence microscopic detection of both ZsGreen1 and tdTomato fluorescent proteins, markers for the transduced BMMSCs, indicated successful anchorage of multiple-transduced BMMSCs in hippocampus CA1 and temporal cortex areas by migration through the network of blood vessels (Figure 2) after the transplantation. These results well reproduced our previous findings.^10, 11^ Nevertheless, the nature of these cells needs to be further characterized, and studies are warranted to show whether engrafts have integrated into the neural network (Figure 3). Importantly, both immunohistochemistry and Western blotting analyses detected significantly increased expression of BDNF and VEGF in the brain with BM-B&V engraftment in contrast to all the other groups (Figure 3). Although overexpression of BDNF and VEGF was not exactly co-localized with the fluorescent markers for transplanted cells here as shown in Figure 2, taken together the results still suggest that BM-B&V engraftment might have markedly boosted levels of BDNF and VEGF in the injured brain. As shown previously, transplantation of naive BMMSCs alleviated brain cell death and improved neurofunctional performance in animals.^10^ Notably, however, once BMMSCs were pre-transduced with lentivirus to overexpress both BDNF and VEGF prior to transplantation, the beneficial efficacy was remarkably enhanced. In this regard, our engineered BMMSCs that co-express high levels of rat BDNF and VEGF not only migrated into and stayed in the ischemia-vulnerable regions (hippocampus and temporal cortex) of the brain, but also maintained overexpression of both BDNF and VEGF *in situ*, and thereby protected brain from ischemic attack. These results extend previous findings.^37, 38, 39^ In addition, immunohistochemistry revealed significantly increased BDNF immunoreactivity in almost all the cells in both hippocampus and temporal cortex areas of the brains in BM-B&V group (Figure 3). As other studies showed lentivirus-transduced MSCs by the similar constructs used here to secrete BDNF or VEGF,^40, 41^ future studies are needed to validate the speculation that overexpressed BDNF from transduced BMMSCs is endocytosed by host neuronal and astroglial cells,^42, 43^ consequently lead to a global increase in BDNF immunoreactivity in most cells as well as the background staining in these brains, and further aids to the brain protection.

*BDNF* gene normally generates a number of alternative transcripts with variable lengths.^44, 45^ BDNF derived from exon-I-containing transcript is widely expressed and is also the dominant isoform in the brain; whereas that from exon IV transcript, which is lack of the codons for the first eight amino-acid residues that are present in exon I, is also one of the common intracellular isoforms and processed normally by convertase, which cleaves BDNF precursor to generate mature BDNF.^46, 47^ It is also known that BDNF plays an extremely important role in the central nervous system through TrkB or P75/SorCS2 receptors to elicit various intracellular signaling pathways, by which contribute to the survival and morphogenesis of neuronal cells as well as the maintenance of homeostatic neuroplasticity.^48, 49, 50^ Exposure of neurons to mature BDNF induces activation of *β*-catenin, ERKs, and Akt pathways to promote neuronal survival by escaping from death challenges such as trophic factor withdrawal or nitric oxide (NO) exposure.^51^ Here we showed that transplantation of BMMSCs with overexpression of BDNF significantly reduced TUNEL-positive degenerating cells in the ischemia-vulnerable regions in the brain, which are in agreement with the aforementioned studies. Similar to BDNF, VEGF is also a crucial player in the process of cell proliferation and inflammation, particularly for angiogenesis. In addition to being a gatekeeper at the neurovascular interface to control the supply of oxygen and nutrients, VEGF also orchestrates brain plasticity with BDNF.^52, 53^ In this study, rat exon IV BDNF along with VEGF-A was efficiently overexpressed in BMMSCs and delivered to the lesioned areas in the brain via cell transplantation through the vein. Although animals were monitored only for 7 days after the CA-GCII and cell transplantation, significant benefits were shown in the group that was transplanted by cells expressing both BDNF and VEGF. Interestingly, administration of exogenous VEGF promotes cerebral angiogenesis to increase the vascular density in the brain parenchyma, but it also causes breakdown of the blood–brain barrier and provokes neuroinflammatory changes in the adult brain or increases severity of brain edema and infarct volume in rodents with brain ischemia,^54, 55^ by contrast delivery of VEGF and BDNF simultaneously through transplantation of engineered BMMSCs significantly reduced brain edema and cell damage in this study. Moreover, enhanced angiogenesis also occurred in the same group of animals in contrast to the blank control as well as the group with naive BMMSC transplants. Newly generated neovasculature in the brain might contribute to improving blood supply to assist the activation of neuroprotective machinery after injury. Importantly, these observations not only demonstrate that both BDNF and VEGF delivered by BMMSCs are functionally active in the injured central nervous system, but they also suggest that their activities might be likely modulated by each other and/or by BMMSCs, possibly through the release of anti-inflammatory and immunomodulatory cytokines.^56^ Nevertheless, further studies are warranted to define the molecular basis relevant to the potential modulatory action of BDNF and BMMSC on VEGF activities.

In conclusion, our study demonstrates the neuroprotective efficacy of engineered BMMSCs that co-overexpress BDNF and VEGF for the treatment of CA-GCII in rats. The results provide a proof of concept for further translational validation of engineered BMMSCs for the treatment of CA-GCII before test in patients in clinic. Additional investigations are warranted to characterize the fate of the engraftment and to assess the therapeutic efficacy in a long-term follow-up, particularly about the effects on the cognitive evolution in addition to the changes in the motor sensory performance after the treatments.

## Materials and methods

### Animals

Immature male Sprague-Dawley (SD) rats (4–5-week old, about 100–150 g body weight) and adult naive male SD rats (about 6-month old, about 280–300 g body weight) were obtained from the Experimental Animal Center of Sun Yat-sen University (Guangzhou, China) and bred in the Guangdong Medical Laboratory Animal Center (Guangzhou, China) for preparation of BMMSCs and establishment of CA-GCII model, respectively. All of the rats used in this study were housed (one per cage) in the animal facility of the Experimental Animal Center of Sun Yat-sen University under a 12 h light/12 h darkness cycle at 22±2 °C with food and water available *ad libitum*. All procedures were approved by the Institutional Animal Care and Use Committee of Sun Yat-Sen University.

### Preparation of BMMSCs, lentiviral constructs, and transduction

Isolation and cultivation of BMMSCs were conducted as previously described with a slight modification.^57^ Briefly, bone marrow cells were collected from the femur and tibia of immature SD rats following killing, and pelleted at 1500 rpm for 5 min. Cells were re-suspended with Dulbecco’s modified Eagle’s medium (Thermo Fisher Scientific, Beijing, China) supplemented with 10% non-heat inactivated fetal bovine serum (Thermo Fisher Scientific) and grown in culture flasks in humidified atmosphere containing 5% CO_2_ at 37 °C. Prior to lentivirus transduction, cells at passage 3 were characterized by flow cytometric analysis using a BD FACSCalibur (BD Biosciences, San Jose, CA, USA) after staining with a fluorescein isothiocyanate-labeled specific antibody against CD34, CD44, CD45, or CD90 (BD Pharmingen, Shanghai, China) to determine the nature of cells.

Lentiviral constructs that express rat BDNF or VEGF were prepared by Land Biology (Guangzhou, China) using Clontech pLVX-IRES-ZsGreen1 and pLVX-IRES-tdTomato bicistronic shuttle vectors (Takara Biomed Technology, Beijing, China). Rat brain BDNF exon IV and VEGF-A cDNAs were prepared by reverse transcription polymerase chain reaction (RT-PCR). Primers for preparing the coding sequence of exon IV BDNF (GeneBank accession number: NM_012513) are: 5′-CCGGAATTCGCCACC ATGACCATCCTTTTCCTTAC-3′ (forward) and 5′-CGCGGATCCCTATCTTCCCC TTTTAATGG-3′ (reverse); and for that of VEGF-A (GeneBank accession number NM_031836) are: 5′-CCGGAATTCGCCACCGT CGCGCTGACGGACAGACAG-3′ (forward) and 5′-CGCGGATTCTCACCGCCTTGGCTTGTCAC-3′ (reverse). Both BDNF (exon IV transcript) and VEGF-A open reading frame sequences were inserted into the vectors through EcoRI and BamHI sites to get pLVX-BDNF-IRES-ZsGreen1 and pLVX-VEGF-IRES-tdTomato constructs. Then human embryonic kidney HEK293T cells in log-phase growth were transfected by sequencing-verified constructs with Lipofectamine 2000 for packaging lentiviral particles. Lentivirus particles were directly collected and concentrated from cell culture media 48 h after transduction by multi-steps of ultracentrifugation (50 000 × *g*, 2 h at 4 °C). Both titers (transduction units, TU) and multiplicity of infection of concentrated lentivirus particles were determined in HEK293T cells grown in 96-well plates by serial dilutions.

BMMSCs were co-transduced with the lentivirus constructs at a multiplicity of infection of 10. Transduction efficiency and expression of BDNF and VEGF were examined 48 h after transduction by fluorescence microscopic visualization of ZsGreen1 and tdTomato fluorescence, and detection of the transcripts and the proteins of interest by RT-qPCR and Western blotting, respectively. Meanwhile, cells were collected and re-suspended in PBS for transplantation.

### Establishment of CA-GCII model, cell transplantation, and post-treatment procedures

The rat CA-GCII model was established on adult SD rats as previously described.^11^ Briefly, surgical procedures were performed to install a Model 90309 multi-channel physiological detector (Spacelabs Medical, North Lauderdal, FL, USA) to monitor the mean arterial pressure, body temperature, and electrocardiogram changes of experimental rats. Vecuronium bromide (Zhejiang Xianju Pharmaceutical, Xianju, China) was given (1 mg/kg body weight, i.v.) to suppress animal respiration until breathing gradually stopped for 10 s. Once no arterial pulse was detected and the mean arterial pressure dropped below 20 mmHg, the animal was monitored for 6 min and then the cardiopulmonary resuscitation was performed immediately by repeated chest compressions with a specially designed device. After 2 min cardiopulmonary resuscitation, epinephrine (0.01 mg in 0.1 ml) and heparin saline (0.5 IU in 0.1 ml) were injected via femoral artery. Meanwhile, mechanical ventilation was applied for 15 min with pure oxygen followed by 50% oxygen for 30 min (tidal volume=0.6 ml/100 g body weight, frequency=100/min) with a specially designed animal ventilator. ROSC was confirmed as the mean arterial pressure maintained above 60 mmHg for more than 5 min. When the spontaneous circulation was fully restored, the animal had experienced in total about 8 min CA. Cell transplantation was performed 2 h after ROSC by direct injection of 500 *μ*l BMMSC suspension (about 3 × 10^6^ cells) via the jugular vein. Animals given sham surgery or CA-GCII with administration of equal volume of PBS or un-transduced naive BMMSCs were used as controls. Accordingly, rats were grouped (*N*=10) as: CA-GCII with BDNF&VEGF-BMMSC transplantation (BM-B&V); CA-GCII with naive BMMSC transplantation (BM-Vehicle); CA-GCII with PBS control (Blank); and sham-operated control (Sham). One week (7 days) after cell transplantation or control treatments, the neurofunctional performance of rats was assessed. Then animals were sedated with CO_2_ gas combined with intraperitoneal injection of pentobarbital (1.0 ml/kg, 4.5% injection solution) and transcardially perfused with physical saline to remove blood residues after the collection of blood (serum) via the inferior vena cava. The brains were rapidly removed; the hippocampus and temporal cortex were dissected from one hemisphere (right side) of each brain for preparation of tissue lysates for Western blotting, whereas the other half hemisphere (left side) of each brain from half of the rats (*N*=5) were used to measure the water content, and those from the other half of the rats (*N*=5) were directly fixed in 10% formalin in 1 × PBS (pH 7.4) overnight at 4 °C followed by dehydration and paraffin embedding for preparing brain sections for neuropathological analyses.

### Neurofunctional assessment

The overall neurofunctional performance of rats was assessed by a comprehensive neurologic deficit score (NDS) at day 7 after cell transplantation according to the published method.^58^ This score system integrates the status of overall consciousness, arousal, respiration, brain stem function, motor, sensory, and activity of seizures of an animal. The results of NDS can range from 80 to 0, indicating from the ‘normal’ to ‘brain dead’ of tested rats, respectively.

### Determination of water content in the brain

Rat brains were weighted immediately after complete removal of the skull and reweighted after subsequent desiccation at 105 °C for 48 h. The brain water content was calculated from: (Weight_wet_–Weight_dry_)/Weight_wet_ × 100%.

### Neuropathological analyses: H&E staining, terminal deoxynucleotidyl transferase (TdT)-mediated dUTP-biotin nick-end labeling (TUNEL), and immunohistochemistry

Paraffin-embedded coronal sections (10 *μ*m thick) were used for all the neuropathological analyses. H&E staining was performed according to the conventional protocol. For TUNEL staining, sections were boiled by microwaving in citrate buffer (10 mM, pH 6.4) for 5 min for antigen retrieval after de-paraffinization and rehydration procedures. Then sections were directly incubated with the TUNEL mix from the *In Situ* Cell Death Detection Kit-POD (Roche Diagnostics, Indianapolis, IN, USA) according to the manufacturer’s protocol. Alternatively, microwave pre-treated sections were incubated with 2% hydrogen peroxide in PBS for 15 min at RT to quench endogenous peroxidase activity followed by 10 min incubation with goat serum to block non-specific binding. Then sections were incubated with a properly diluted primary antibody (rabbit polyclonal anti-BDNF, 1:100; rabbit polyclonal anti-VEGF, 1:200; mouse monoclonal anti-RECA-1, 1:500; all three antibodies were purchased from Abcam, Shanghai, China) overnight at 4 °C. Immunoreactivity was detected using the ABC protocol followed by staining with diaminobenzidine (Boster, Wuhan, China). All the sections were briefly counterstained to show cell nuclei with hematoxylin, cover slipped, and examined under light microscopy. BDNF-/VEGF- or TUNEL-positive cells or pyknotic cells in H&E *versus* total numbers of cells were separately examined/counted from total five non-overlapped microscopic fields within the CA1 region of hippocampus as well as temporal cortex at × 400 magnification, whereas the microvessels (RECA-1-positive structures) were examined within the temporal cortex under × 100 magnification. The immunoreactivity of both BDNF and VEGF was scored according to Remmele and Stegner’s method in both hippocampus and temporal cortex areas as described,^11, 59^ and the microvessel density was calculated as previously described.^60^

### Real-time RT-qPCR

Total RNA was extracted from BMMSCs using Trizol (Thermo Fisher Scientific) and 1.0 *μ*g total RNA was reverse transcribed using the M-MLV reverse transcriptase system (Promega, Madison, WI, USA) according to the manufacturers’ instructions. qPCR was performed with a total volume of 20 *μ*l, which included 5 *μ*l diluted (1 : 20) cDNA mixture, forward primer 0.5 *μ*l (10 *μ*M), reverse primer 0.5 *μ*l (10 *μ*M), 2 × SYBR Green qPCR SuperMix (TOYOBO, Osaka, Japan) 10 *μ*l, and PCR water 4 *μ*l. The reaction was subjected to 40-cycle amplification (95 °C, 5 min; 95 °C, 15 s; 60 °C, 15 s; 72 °C, 32 s). Primers for BDNF were: 5′-GGGTCACAGCGGCAGATAAA-3′ (forward), 5′-CGATTGGGTA GTTCGGCATT-3′ (reverse); and VEGF were: 5′-CACTGGACCCTGGCTTTACT-3′ (forward), and 5′-TCAATTGGACGGCAATAGCT-3′ (reverse). The 18SrRNA was used as an internal reference, and its PCR primers were: 5′-CCTGGATACCGCAG CTAGGA-3′ (forward), and 5′-GCGGCGCAATACGAATGCCCC-3′ (reverse). The abundance of the interest genes was calculated based on ΔΔCT and depicted as 2^ΔΔCT^.

### Western blotting

Western blotting was conducted as described previously.^61^ Briefly, dissected hippocampus and cortex tissues or cultured BMMSCs were homogenized in 500 *μ*l RIPA lysis buffer mixed with protease inhibitor cocktail (Sigma-Aldrich, St. Louis, MO, USA). The resultant supernatants of tissue/cell lysates after removal of pellets by centrifugation (14 000 rpm × 10 min) were measured for protein concentration using the BCA total protein assay kit (Keygen Biotech, Nanjing, China). Then 20 *μ*g of total protein lysate from each sample was resolved by 10% sodium dodecyl sulfate-polyacrylamide gel electrophoresis and transferred to a PVDF membrane (Millipore, Billerica, MA, USA) under 100 volts for about 1 h at 4 °C. The membranes were then blocked in Blotto solution (8% defat milk in 1 × TBST) for 1 h, and incubated with a properly diluted rabbit polyclonal anti-BDNF IgG (1:500, Abcam) or rabbit polyclonal anti-VEGF IgG (1:1000, Abcam) in TBST-diluted 10% Blotto overnight at 4 °C and a horseradish peroxidase-conjugated anti-rabbit IgG secondary antibody (1:2000, 1 h at RT). The results were visualized by ECL chemiluminescence kit and X-ray film exposure. The house-keeping gene product, GAPDH, was used as a loading control for brain tissues as well as whole-cell lysates. HRP-conjugated mouse monoclonal IgG against GAPDH (1:10 000, KangChen Biotech, Shanghai, China) was used for immune-blotting. The blots were quantified by densitometry using Quantity One software (BioRad, Hercules, CA, USA). Measurements were normalized to the corresponding GAPDH loading control and depicted as relative abundance.

### Determination of S100B by enzyme-linked immunosorbent assay (ELISA)

The levels of S100B protein in the serum were determined using a commercial ELISA kit (CUSABIO Life Science, Wuhan, China) according to the manufacturer’s instruction.

### Statistical analysis

All assays were performed in triplicate or up to five replicas. Statistical analysis was performed using IBM SPSS Statistics 19.0 software. Student’s *t*-test was performed to test the significant difference between groups. Results were depicted as means±S.D. All categorical data from the NDS assessment and immunoreactivity scores of both BDNF and VEGF were analyzed using Mann–Whitney–Wilcoxon test. The values of *P*<0.05 were considered as statistically significant.
